# Engraftment of Bacteria after Fecal Microbiota Transplantation Is Dependent on Both Frequency of Dosing and Duration of Preparative Antibiotic Regimen

**DOI:** 10.3390/microorganisms9071399

**Published:** 2021-06-29

**Authors:** Vancheswaran Gopalakrishnan, Elizabeth Ashley Dozier, Matthew S. Glover, Steven Novick, Michael Ford, Christopher Morehouse, Paul Warrener, Carolina Caceres, Sonja Hess, Bret R. Sellman, Taylor S. Cohen

**Affiliations:** 1 Microbiome Discovery, Microbial Sciences, BioPharmaceuticals R & D, AstraZeneca, Gaithersburg, MD 20878, USA; vancheswaran.gopalakrishnan@astrazeneca.com (V.G.); elizabeth.dozier@astrazeneca.com (E.A.D.); christopher.morehouse@astrazeneca.com (C.M.); paul.warrener@astrazeneca.com (P.W.); carolina.caceres@astrazeneca.com (C.C.); bret.sellman@astrazeneca.com (B.R.S.); 2 Dynamic Omics, Antibody Discovery & Protein Engineering, R & D, AstraZeneca, Gaithersburg, MD 20878, USA; matthew.glover@astrazeneca.com (M.S.G.); sonja.hess@astrazeneca.com (S.H.); 3 Data Sciences and Quantitative Biology, Discovery Sciences, BioPharmaceuticals R & D, AstraZeneca, Gaithersburg, MD 20878, USA; steven.novick@astrazeneca.com; 4 Animal Sciences and Technologies, R & D, AstraZeneca, Gaithersburg, MD 20878, USA; michael.ford1@astrazeneca.com

**Keywords:** microbiome, conventional mice, antibiotics, engraftment

## Abstract

The gut microbiota has emerged as a key mediator of human physiology, and germ-free mice have been essential in demonstrating a role for the microbiome in disease. Preclinical models using conventional mice offer the advantage of working with a mature immune system. However, optimal protocols for fecal microbiota transplant (FMT) engraftment in conventional mice are yet to be established. Conventional BALB/c mice were randomized to receive 3-day (3d) or 3-week (3w) antibiotic (ABX) regimen in their drinking water followed by 1 or 5-daily FMTs from a human donor. Fecal samples were collected longitudinally and characterized using 16S ribosomal RNA (rRNA) sequencing. Semi-targeted metabolomic profiling of fecal samples was also done with liquid chromatography–mass spectrometry (LC-MS). Lastly, we sought to confirm our findings in BKS mice. Recovery of baseline diversity scores were greatest in the 3d groups, driven by re-emergence of mouse commensal microbiota, whereas the most resemblance to donor microbiota was seen in the 3w + 5-FMT group. Amplicon sequence variants (ASVs) that were linked to the input material (human ASVs) engrafted to a significantly greater extent when compared to mouse ASVs in the 3-week groups but not the 3-day groups. Lastly, comparison of metabolomic profiles revealed distinct functional profiles by ABX regimen. These results indicate successful model optimization and emphasize the importance of ABX duration and frequency of FMT dosing; the most stable and reliable colonization by donor ASVs was seen in the 3wk + 5-FMT group.

## 1. Introduction

The gastrointestinal tract is composed of a complex microbial community that plays a critical role in several key host functions [1]. Accumulating evidence has linked the gut microbiome to changes in host physiology mediated through the immune system; alterations in microbial equilibrium often referred to as ‘dysbiosis’ has been associated with a wide range of disease conditions [2,3,4]. This is particularly relevant in light of several recent clinical trials showing that modulation of the gut microbiome via fecal microbiota transplant (FMT) or tailored bacterial consortia can improve treatment responses across various diseases ranging from cancer [5,6,7], recurrent *Clostridioides difficile* infection [8], to ulcerative colitis [9]. While the utility of microbial modulation in improving clinical outcomes in patients continues to be evaluated in clinical trials, its mechanistic underpinnings are still incompletely understood. Therefore, translational studies in animal models are necessary to begin to transition from association to causation.

Germ-free mice have long been the cornerstone for translational research in microbiome studies and have been used to demonstrate causal relationships by transferring the donor phenotype following microbiome engraftment [10,11]. They are devoid of any endogenous microbiota and can be colonized by foreign microbiota derived from fresh or frozen human or murine stool samples. They offer the advantage of allowing large proportions of the input microbiota to engraft, thereby enabling greater humanization of the recipient mice [12,13]. Nevertheless, there are several challenges associated with using this system. As a consequence of not containing any microbiota during development, germ-free mice have altered metabolic capabilities, an immature gut with a thin mucus layer [14] and an immune system characterized by atrophic Peyer’s patches, reduced B and T-cell numbers and reduced numbers of IgA secreting plasma cells [15,16]. Transfer of foreign microbiota triggers developmental changes in the germ-free recipients which may mask disease effects. Furthermore, the handling of germ-free mice is logistically challenging and technique sensitive as these mice are quite susceptible to contamination [17].

Preclinical animal models using conventional, specific pathogen free (SPF) mice offer the advantage of working with an intact and mature immune system, and a greater context for studying host–microbiota interactions [18,19]. This model involves preconditioning the gastrointestinal tracts (gut) of recipient mice with antibiotics (ABX) followed by the introduction of candidate bacteria. Accordingly, broad-spectrum ABX have been shown to result in better engraftment compared to single ABX [20], though several other factors play a role, such as richness of input material [21] and the presence of any residual ABX; bowel cleansing with polyethylene glycol (PEG) has also yielded inconsistent results [22]. ABX cleanout of conventional mice is particularly complicated by re-emergence of baseline mouse microbiota [19]. Therefore, optimal protocols for microbial engraftment from foreign input material such as stool into conventional mice are yet to be established.

Herein we compare and contrast the impact of different antibiotic regimens, and frequency of fecal microbiota transplant (FMT) dosing on engraftment of bacteria in conventional mice and assess its durability over time. We evaluated the engraftment kinetics of microbiota that were linked to the input material and those that were unique to the recipient mice at baseline. We also investigated functional changes using semi-targeted metabolomics.

## 2. Materials and Methods

### 2.1. Experimental Model

Animal experiments were carried out in replicate with conventional specific pathogen free (SPF), female BALB/c mice at 6 weeks of age, purchased from Envigo and housed at AstraZeneca in Techniplast Bioexclusion cages (air-tight, positive pressure, cage-level hepa filter). Two independent donors were randomly chosen for each experiment. All procedures were approved by and performed in accordance to The Institutional Animal Care and Use Committee at AstraZeneca (AZ).

**Figure 1:**
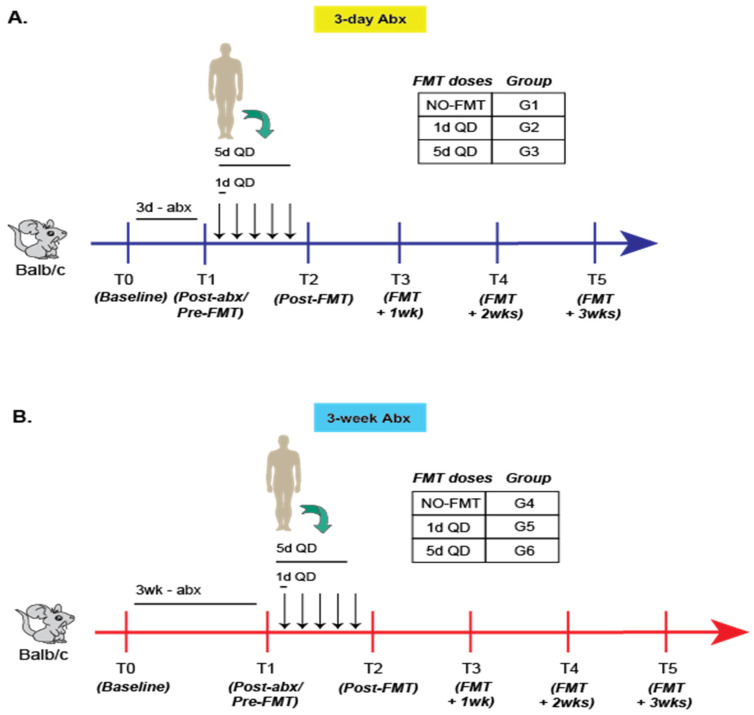
Experimental schema for primary experiment in Balb/c mice. Classification and sampling scheme for mice (*n* = 5/group) receiving either the (**A**) 3-day or (**B**) 3-week antibiotic regimen. Representative data from two experiments shown.

Mice were randomly assigned into 6 groups with 5 mice per group (Figure 1). Groups (G) 1–3 received our 3-day (3d) ABX regimen, wherein G1 received no FMT, G2 received 1 fecal FMT dose (3d + 1-FMT), and G3 5 FMT doses (3d + 5-FMT). G 4–6 received our 3-week ABX regimen (3wk), wherein G4 received no FMT, G5 received 1 FMT dose (3wk + 1-FMT), and G6 received 5 FMT (3-wk + 5-FMT) doses.

#### Antibiotic Regimen

The 3-day ABX regimen [23] was a cocktail consisting of 0.2 mg/mL neomycin sulfate, 0.1 mg/mL vancomycin and 1g aspartame in sterile water administered ad libitum within a bioexclusion cage water bottle. The antibiotic cocktail was administered for 3 sequential days, immediately followed by 12 h of 10% PEG in sterile water to flush antibiotic cocktail from gut.

The 3-week ABX regimen [18] consisted of specific antibiotic cocktails administered ad libitum in bioexclusion cage water bottles in a cycled manner, with each cycle consisting of 5 sequential days of an antibiotic cocktail followed by a 2 day water wash-out.

Cycle 1 is the non-absorbable antibiotic cocktail, each at 1 mg/mL consisting of ertapenem sodium (Invanz^®^; Merck and Co., Inc., Kenilworth, NJ, USA), neomycin sulfate (MilliporeSigma, Burlington, VT, USA), and vancomycin hydrochloride (BioVision Incorporated, Milpitas, CA, USA).

Cycle 2 is the absorbable antibiotic cocktail, each at 1 mg/mL, consisting of ampicillin, cefoperazone sodium salt, and clindamycin hydrochloride (MilliporeSigma).

Cycle 3 is the non-absorbable antibiotic cocktail, each at 1 mg/mL, consisting of ertapenem sodium (Invanz^®^; Merck and Co., Inc.), neomycin sulfate (MilliporeSigma), and vancomycin hydrochloride (BioVision Incorporated).

All antibiotic cocktails were prepared on the first day of the cycle in sterile DNase/RNase-free molecular grade water.

### 2.2. FMT Preparation and Gavage

Donors were randomly chosen from an internal repository of stool samples at AZ. Aliquoted human stool samples stored at −80 °C were thawed on wet ice. Fecal suspensions were prepared at 10 mg/mL in sterile 1X phosphate-buffered saline (PBS), homogenized for 2 cycles at 30 Hz for 60 s a cycle with the Lysis Matrix A tubes (MP Biomedicals, Irvine, CA, USA), then filtered 2X with a 100 μm followed by a 40 μm cell strainer to remove any unhomogenized particulates. A total of 0.2 mL of FMT preparations were administered to mice by oral gavage immediately after preparation [11] under aseptic conditions using a laminar flow hood.

### 2.3. DNA Extraction

DNA extraction of 10–50 mg of fecal sample was performed using the QIAamp 96 PowerFecal QIAcube HT Kit (Qiagen, Hilden, Germany) according to the manufacturer’s instructions. Briefly, fecal samples were added to Power Bead tubes (Garnet, 0.7 mm, Qiagen) containing 800 μL prewarmed Buffer PW1 and homogenized by mechanical disruption in a TissueLyser II for 10 min at 30 Hz. Samples were subsequently centrifuged at 6000 RPM for 5 min and 400 μL of supernatant was transferred into a deep 96-well block. Then, 150 μL of buffer C3 was added and mixed thoroughly by pipetting. Samples were incubated overnight at 4 °C followed by centrifugation at 3500 RPM for 10 min. In total, 300 μL of this supernatant was transferred to an S block containing 20 µL of proteinase K followed by a 10-min incubation at room temperature. The subsequent steps were carried out on the QIAcube HT running the QIAamp 96 PowerFecal protocol. DNA was eluted in a final volume of 100 µL.

### 2.4. 16S rRNA Sequencing

#### 2.4.1. Amplicon PCR and Library Preparation

The Amplicon polymerase chain reaction (PCR) and library preparation was carried out essentially as described before [24]. Briefly, V4 16S rRNA gene amplicons were constructed using dual-index primers and AccuPrime Taq DNA Polymerase, High Fidelity (Invitrogen, Waltham, MA, USA). Amplicon clean up and normalization was performed using the SequalPrep Normalization Kit (Invitrogen). Amplicons were pooled and the final library concentration was determined by quantitative PCR using the NEBNext Library Quant Kit (New England Biolabs, Ipswich, MA, USA).

#### 2.4.2. Sequencing

Libraries were mixed with PhiX Control v3 (Illumina, San Diego, CA, USA) and denatured using fresh NaOH. Pooled libraries were sequenced on an Illumina MiSeq^®^ instrument using a 2 × 250 base-pair (bp) MiSeq^®^ Reagent Kit v2.

#### 2.4.3. Analysis

After appropriate read merging and quality filtering, denoising of V4 amplicons into amplicon sequence variants (ASVs) was done with DADA2 [25]. Phylogenetic classification of ASVs and calculation of level abundances was done by mapping to SILVA v132 [26]. Quality plots of Phred scores and error plots were well within acceptable limits (Figures S1–S3). In order to track ‘origin’, ASVs were classified as (1) human: if they were identified only in the input FMT material, (2) mouse: if they were identified in any mouse from a group at baseline and (3) both: if they were common to both (1) and (2) above. Lastly, estimation of alpha (Inverse Simpson) and beta diversity (Weighted and Unweighted UniFrac; [27]) was done using QIIME (Quantitative Insights into Microbial Ecology) [28].

### 2.5. Metabolomics

Analysis of short-chain fatty acids (SCFAs) in fecal samples was performed based on a method developed by Borchers and coworkers [29]. This method was previously modified for semitargeted analysis by liquid chromatography–mass spectrometry (LC-MS) and described in detail [30]. Metabolite extraction of fecal samples was performed with 50:50 acetonitrile:water at a ratio of 10 µL extraction solvent:1 mg fecal sample. Samples were manually homogenized, mixed at 2000 revolutions per minute (RPM) for 20 min at 4 °C, and centrifuged for 15 min at 18,000× *g* and 4 °C. Metabolite extracts (25 µL) were derivatized with 3-nitrophenylhydrazine (3-NPH). Extracts were mixed with 10 µL of 200 mM 3-NPH·HCl in 50:50 acetonitrile:water (ACN:H2O) solution and 10 µL of 120 mM *N*-(3-Dimethylaminopropyl)-*N*′-ethylcarbodiimide·HCl (EDC) in 50:44:6 ACN:H2O:pyridine solution. Derivatization was performed at 40 °C with 800 RPM mixing for 30 min. Derivatization was quenched by addition of 960 µL of 50:50 ACN:H2O at 4 °C.

Relative quantitation of SCFAs was performed by LC-MS on an Agilent 1290 Infinity II LC coupled to a 6560 Q-TOF operated in negative ionization mode as previously described in detail [30]. Briefly, chromatography was performed with a Waters Acquity UPLC BEH C18 column (1.7 µm, 2.1 mm × 100 mm). Mobile phases A and B were composed of 0.01% formic acid in H20 and 0.01% formic acid in ACN, respectively.

Feature extraction, retention time alignment, and relative quantitation of SCFAs was performed using MS-DIAL version 4.60 [31]. Identities of SCFAs were verified by *m*/*z* and retention time matching using pure 3-NPH-derivatized SCFA standards. SCFA abundances were normalized in MS-DIAL to the total ion chromatogram (TIC) in order to account for any differences in metabolite content of fecal samples from different groups. Normalized abundances are reported in Table S1. Donor values from 3 replicates were averaged.

### 2.6. Statistical Analysis

Comparison of alpha diversity scores between groups was performed using the Kruskal–Wallis test. Ordination of beta-diversity distances were examined using principal coordinate analysis (PCoA) and centroids were compared by Analysis of Similarity (ANOSIM) [32]. In tracking engraftment kinetics, human and mouse derived ASV abundances at timepoints T3–T5 were compared using the one-sided paired Wilcoxon signed rank test with the alternative hypothesis that human-derived ASVs will occupy more of the microbial niche compared to mouse ASVs after FMT. Lastly, principal component analysis (PCA) was used to compare the metabolic potential of G3 versus G6 mice. All statistical analyses and visualizations (using the ggplot2 package [33]) were done in R [34].

## 3. Results

All mice were randomized to receive 3-day (3d) or 3-week (3w) ABX followed by 0, 1 or 5 doses of FMT. Fecal samples were collected longitudinally at prespecified timepoints (T) and characterized using 16S rRNA sequencing (Figure 1, also see Section 2).

### 3.1. Longitudinal Changes in Alpha and Beta-Diversity Are Appreciable in All Groups

**Figure 2:**
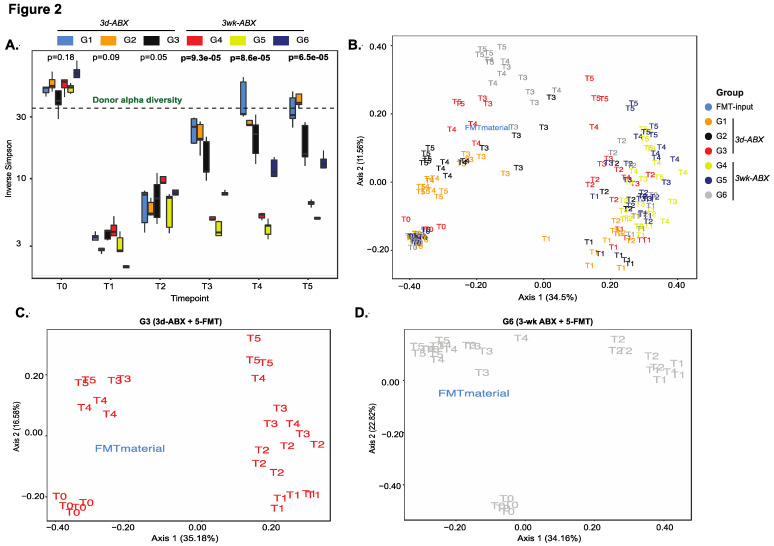
Analysis of diversity. (**A**) Box plots of longitudinal changes in alpha diversity scores over time (*n* = 5 mice/group; *p*-value by Kruskal–Wallis test). PCoA plot of unweighted UniFrac beta-diversity distances in (**B**) all groups, (**C**) G3 mice and (**D**) G6 mice (*n* = 5/group; *p*-value by ANOSIM). Other groups in Figure S4.

Overall, there was a significant decline in diversity scores (both alpha diversity, Figure 2A, and richness, Figure S4A) after treatment with antibiotics, (T1–T2), with a steady increase thereafter (T2–T5) in all groups. Global differences between groups in both richness and alpha-diversity were best appreciated in the weeks following FMT dosing (T3–T5). Mice in the 3d ABX groups (G1–G3) recovered most of their baseline diversity scores over time, even beyond the level of the input FMT material (Figure 2A and Figure S4A). Conversely, diversity of the microbiome in mice in the 3w ABX groups (G4–G6) remained below baseline for the remainder of the experiment.

On tracking compositional differences over time using unweighted UniFrac distances, we found notable shifts in sample clusters longitudinally. Expectedly, the most profound effect was seen after antibiotic treatment. The microbiomes of mice in G1 and G2 almost completely reverted back to their baseline state, with one FMT dose in G2 proving unable to find a niche within the endogenous microbiota (Figure 2B and Figure S4B,C). Mice in G4 received no FMT after 3 w-ABX and showed least resemblance to the donor or to their own baseline state (Figure S4D). Importantly, the greatest resemblance to the donor was seen in mice receiving five doses of the FMT (G3 and G6), and more so in G6 (Figure 2B–D).

### 3.2. Greater Engraftment Seen in Mice Receiving 3wABX Compared to Those Receiving 3d ABX

In order to track kinetics of engraftment, ASVs within a group were classified as ‘human’ ASVs if they were linked to the input FMT material, and ‘mouse’ ASVs if they were only identified in any one of the recipient mice at baseline. ASVs that were shared between the input human material and the baseline mouse microbiome were also included in the human ASVs based on the assumption that foreign bacteria may thrive in the presence of similar species in the recipient [35].

**Figure 3:**
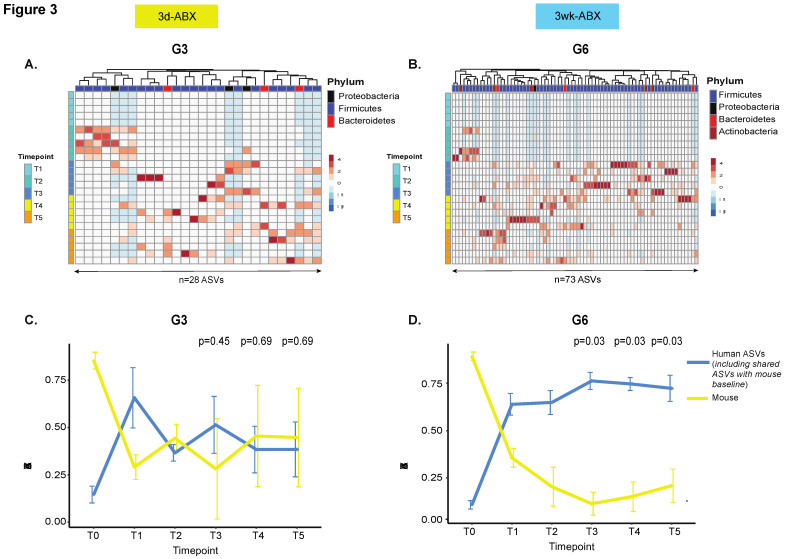
Comparison of engraftment kinetics. Unsupervised hierarchical clustering heatmap of ASVs uniquely identified in the input FMT material in (**A**) G3 and (**B**) G6 mice (*n* = 5 mice/group). Columns represent ASVs. Rows are samples temporally arranged from T1 through T5. Line plot to compare changes in ‘human’ and ‘mouse’ ASV abundances over time in (**C**) G3 and (**D**) G6 mice (*n* = 5 mice/group). The mean and standard deviation of ASV abundances is represented at each time point. The *p*-value is calculated from a one-sided paired Wilcoxon signed-rank test and adjusted for false discovery rate [36]. Other groups in Figure S5. Additionally, see line plot for ASVs uniquely derived from input material in Figure S6.

Among the groups that received five doses of FMT, more robust engraftment of ASVs uniquely identified in the input FMT material was seen in G6 compared to G3 (*n* = 73 ASVs in G6 versus *n* = 28 in G3). In both G3 and G6 mice, the greatest density of engraftment was seen at T3 (1-week post-FMT), with a successive decline thereafter (Figure 3A,B). In order to track longitudinal changes in ASV abundances by origin we compared mouse ASVs to human ASVs (Figure 3C,D and Figure S5), including those that were uniquely derived from the input material (Figure S6). Re-emergence of mouse ASVs after FMT (T2–T5) was greater in the 3d ABX groups (G2 and G3; Figure 3C, Figures S5A and S6A,B). In these groups, mouse ASVs constituted roughly 50% of the microbial niche at T4 and T5 after 5-FMT doses (G3, Figure 3C and Figure S6B), but more than 75% after only one dose (Figures S5A and S6A). In contrast, in groups that received the 3w ABX regimen, re-emergence of mouse ASVs was less than 25% and occupied significantly lesser amounts of the niche compared to human ASVs (Figure 3D, Figures S5B and S6C,D).

Human ASVs colonized more 60% of the niche after one FMT dose in all groups but dropped to under 50% in G3 (after five FMT doses) and under 25% in G2 (after only one FMT dose) (Figure 3C and Figure S5A). In mice receiving the 3w ABX regimen, human ASVs colonized up to 75% of the niche after FMT, with stable engraftment seen throughout the course of the experiment (Figure 3D and Figure S5B), including ASVs that were uniquely derived from the input material (Figure S6C,D). Our findings were confirmed in a repeat experiment.

### 3.3. Taxonomic and Functional Landscape of Recipient Mice Is Dictated by Choice of ABX Regimen

**Figure 4:**
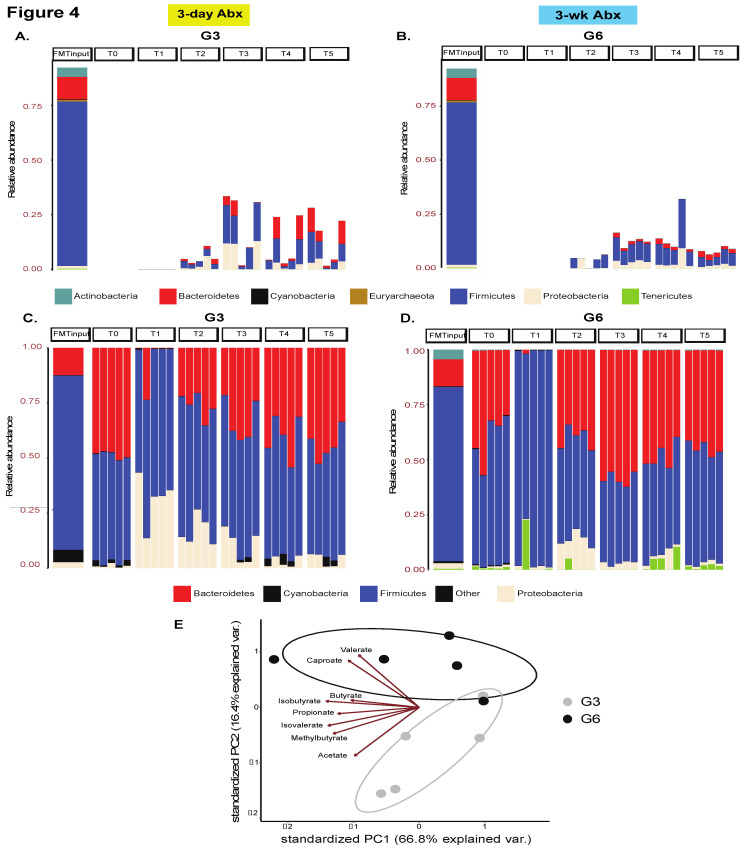
Taxonomic landscape. Stacked bar plot showing engraftment of phyla uniquely derived from the input material in recipient mice in (**A**) G3 and (**B**) G6 mice, *n* = 5 mice/group. See other groups in Figure S7. Additionally, see analogous plot for families in Figure S8. Stacked bar plot comparing the overall taxonomic landscape at the phylum level of the input material and recipient mice over time in (**C**) G3 and (**D**) G6, *n* = 5 mice/group. Only the most abundant taxa are shown. See other groups in Figure S9. See analogous plot for families in Figures S10 and S11. (**E**) Principal component analysis plot of metabolite abundances. Data points represent individual samples (*n* = 5 mice/group) and arrows show an overlaid biplot of loadings for each metabolite.

Next, we characterized the taxonomic distribution across all recipient mice and input FMT material and found that the donor had an enrichment of the Firmicutes phylum and Lachnospiraceae family (Figure 4, Figures S7–S11). Upon tracking the engraftment of taxa that were uniquely derived from the input material, we found greater colonization by Firmicutes and Lachnospiraceae in groups receiving five FMT doses—G3 and G6 (Figure 4A,B and Figure S8B,D). Mice receiving only one FMT dose showed greater colonization by input-derived Proteobacteria and Burkholderiaceae; Proteobacteria were more durably engrafted in G5 (3w ABX) (Figures S7 and S8A,C).

We next considered the overall taxonomic distribution in all mice including taxa that were inherently present in the recipient mice at baseline. Notably, across all groups, there was an increase in Proteobacteria abundance immediately after ABX-cleanout (T1), though eventually they were retained at T4 and T5 only in mice from G5, whereas G2, G3, G4, and G6 mice were colonized instead by Firmicutes and Bacteroidetes (Figure 4C,D and Figure S9B,D). In mice receiving 3d ABX without any subsequent FMT (G1), the Proteobacteria was almost completely replaced by Firmicutes and Bacteroidetes over the course of the experiment (Figure S9A).

Further investigation of all taxa (including those that were human derived) at the family level also revealed important trends. Specifically, at T0 (baseline), all mice were colonized by Lachnospiraceae, followed by Rikenellaceae, Ruminococcaceae, Lactobacillaceae and Bacteroidaceae in order of decreasing abundance (Figures S10 and S11). However, their eventual microbial niche was highly dependent on both the ABX-regimen and number of FMT doses. Among the 3d ABX groups, G1 mice most closely resembled the baseline distribution followed by G2 (Figure S10A,B); G3 mice showed an increase in Rikenellaceae and Tannerellaceae (Figure S10C). In stark contrast, the 3-wk ABX mice showed a major compensatory increase in Bacteroidaceae and Tannerellaceae at the expense of Rikenellaceae (Figure S11A–C).

Importantly, we also performed functional profiling of fecal samples obtained at T5 (3-wk after FMT) from G3 and G6 recipient mice using semi-targeted metabolomics. In these studies, we observed a notable clustering effect by group, suggestive of functional differences, with the biplot indicating that caproate and valerate were driving clustering of G6 whereas acetate and 2-methylbutyrate were driving the clustering of G3 mice. These data suggest that choice of ABX regimen and the type of subsequent engraftment also impacts the metabolic capabilities of the microbiome in recipient mice (Figure 4E, Table S1).

### 3.4. Confirmation of Findings in BKS Mice

**Figure 5:**
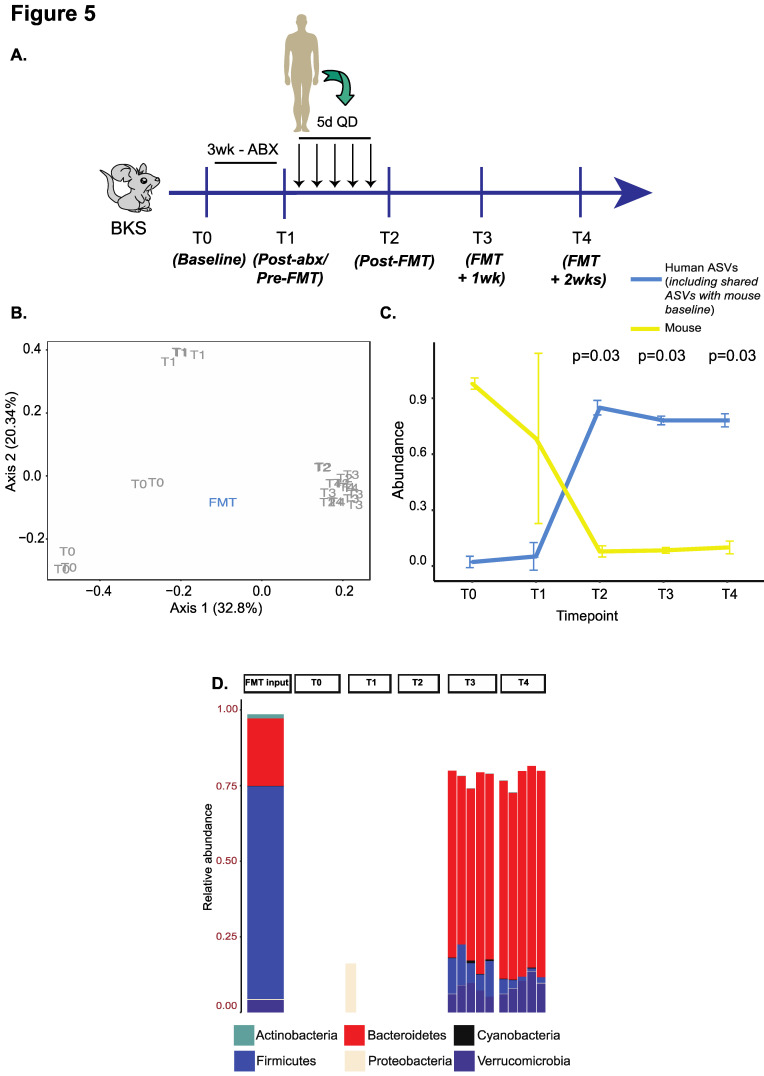
Validation experiment in BKS mice using the G6 regimen. (**A**) Experimental schema. (**B**) PCOA plot of unweighted UniFrac beta-diversity distances with *p*-values calculated by ANOSIM. (**C**) Line plot to compare changes in ‘human’ and ‘mouse’ ASV abundances over time. The *p*-value is calculated from a one-sided paired Wilcoxon signed-rank test and adjusted for false discovery rate. (**D**) Stacked bar plot showing engraftment of phyla uniquely derived from the input material in recipient mice.

Lastly, we sought to confirm if our findings were applicable to mice from other strains such as BKS (Figure 5A), which were treated with the 3w ABX regimen followed by gavage with five doses of FMT (analogous to G6 above) from an unrelated donor. Consistent with our prior findings using beta-diversity, we noted a shift from baseline following ABX treatment. Additionally, there was greater intra-group clustering and a close resemblance to the overall composition of the input FMT material in recipient mice from T2–T4 (Figure 5B). Importantly, mouse ASVs were depleted significantly and re-emerged to only low levels after FMT (<25%) (Figure 5C), suggesting durable engraftment. On characterizing bacterial taxonomy, we observed high levels of Bacteroidetes (and Bacteroidaceae) at post-FMT timepoints in recipient mice that were uniquely derived from the input material at the expense of Firmicutes (and Lachnospiraceae) which was prevalent at baseline (Figure 5D and Figure S12).

## 4. Discussion

Alterations of microbial ecology have been linked to various disease conditions. As such, microbiome modulation strategies are increasingly being considered as an investigational treatment, either as a stand-alone therapy or as an adjunct to improve the efficacy and tolerability of standard of care therapies [5,6,7,37,38]. This paradigm has been primarily spurred by promising results seen with recurrent *Clostridioides difficile* infection which serves as the prototypical example, wherein FMT has achieved high efficacy rates. Clinical resolution has been attributed to shifting of the disease-linked microbiome state and an increased diversity post transplantation [39,40]. More recently, positive outcomes have also been seen in the context of immune-checkpoint blockade therapies [5,6], even in patients who were historically refractory such therapies.

The basic principle of FMT involves transplanting the entire microbial ecosystem of the donor to achieve robust engraftment and reversal of clinical phenotype in recipients. Recent studies have emphasized the importance of establishing donor-derived bacteria alongside the endogenous microbes in the host [41,42]. Establishing a robust preclinical model system that incorporates human-derived microbiota provides the best opportunity to further characterize physiological roles of these bacteria.

Germ-free mice have long been considered the ideal setting to study the physiologic effects of human-derived microbiota on the host, but intrinsic limitations such as incomplete maturation of the intestinal immune system and failure to develop colonization resistance against some pathogens post-transplant of human fecal matter (as opposed to mouse fecal matter) warrant consideration of alternative options [43,44]. Therefore, there is a need for the development conventional mouse models suitable for exploration of FMT and elucidation of mechanisms connecting the microbiome and host.

Use of antibiotics for elimination of host commensals is dependent on several factors such as class, absorption and bioavailability [45]. Therefore, it is improbable for a single antibiotic to achieve high cleanout efficiency against a complex microbial ecosystem, suggesting the need for carefully selected ABX cocktails that can act in synergy [18,46,47]. In this context, it is desirable that the ABX achieves disruption of the existing host microbial community and/or reduction of their density to enable foreign bacteria to durably engraft. It is also important to note, that metrics for successful colonization have not been universally agreed upon with both establishment of diversity and/or engraftment of the dominant bacterial group considered as appropriate [12]. The primary objective is to consider the limitations of the model while generalizing the results of humanization of mouse microbiota [48].

In our study, we characterized ASVs to enhance precision, and longitudinally track exact sequences. Our approach to quantifying engraftment was multipronged. We considered human ASVs that were linked to the donor input material and mouse ASVs that were only identified in the recipient mice at baseline. Successful engraftment required robust acceptance of input ASVs as well as limited re-emergence of mouse ASVs in order to sustain the newly acquired bacteria from the donor. We also tracked a subgroup of ASVs within the human ASVs that were unique to the input material.

We used our ASV classification scheme to compare and contrast two previously established antibiotic schedules [18,23], and examined engraftment of bacteria in two different mouse strains. Both regimens resulted in a dramatic decline in diversity, and an emergence of Proteobacteria after ABX treatment. Following FMT, there was a rise in diversity caused by early colonization with donor bacteria.

We found that pretreatment with the 3w ABX regimen, consisting of three courses of alternating antibiotic cocktails, followed by five doses of FMT (group G6) helped achieve greater resemblance to the input donor material in the recipient mice. These groups had robust and durable engraftment of ASVs derived from the input material and baseline mouse microbiota repopulated the gut ecology to a much lesser extent when compared to mice receiving the 3d ABX regimen (G2 and G3). It is likely that the low levels of re-emergence of pretransplant baseline bacteria in G5 and G6 mice helped create an environment that was permissive towards human ASVs. Consequently, due to the reduced competition from mouse ASVs, shared ASVs (between human and mouse) in BALB/c mice—which originally constituted a small proportion (<10%) of the baseline niche—and human ASVs in BKS mice, thrived considerably in the 3w groups after FMT. This finding is consistent with FMT studies in humans where donor and recipient strains displayed durable coexistence after transplantation [35] suggesting that foreign bacteria can be readily accepted in an environment where similar species/strains are already persisting. It is also clear, that host genetics plays an important role, in determining microbial profiles, and therefore the applicability of our findings in two independent strains of mice is notable [49]. Taken together, our findings suggest that the 3-wk ABX regimen resulted in a greater acceptance of donor bacteria and a robust microbiome shift, which emphasizes the translational relevance of this methodology.

While human and mouse microbiota both share major microbial phyla, namely Firmicutes and Bacteroidetes, they have been shown to differ significantly at lower levels [50,51], and there is a well-documented discordance in the Firmicutes:Bacteroidetes ratio. In both strains of mice (and especially in the BKS mice) there was a preferential increase in abundance of bacteria belonging to the Bacteroidetes phylum at the expense of the Firmicutes phylum, likely linked to the enhanced ability of Bacteroidetes bacteria to extract benefit from mouse chow and the glycoproteins in the intestinal mucus of mice [12,20,52]. Therefore, taxonomic implications may not be directly generalizable to humans. Both ABX regimen and FMT dosing dictated the taxonomic composition of recipient mice, with multiple inoculations resulting in greater resemblance to the donor as was previously reported [20].

There was an outgrowth of Proteobacteria immediately after ABX in all groups, but this was more apparent after treatment with the 3d ABX regimen. Nevertheless, they persisted at post-FMT timepoints only in G5 mice, driven by Proteobacteria ASVs that were derived from the input material. Interestingly, G6 mice, which received more doses of FMT did not retain Proteobacteria and were instead colonized by Firmicutes ASVs from the input material, thereby resulting in these recipients resembling the donor microbiota profile more closely.

Through our semi-targeted metabolomics assay, we found that there were substantial changes in the metabolomes of mice, and that metabolic profiles differed by ABX-regimen consistent with reports from other investigators [53]. Additionally, the identified metabolites were consistent with what would be expected based on the taxonomic landscape in the recipient mice; e.g., Lachnospiraceae, Ruminococcaceae and butyrate production [54]. This is also a relevant finding in the context of being able to gain insight into the dynamic interplay between microbiome, disease and therapy.

## 5. Conclusions

FMT has gained popularity as a therapeutic modality for various disease conditions, though the engraftment kinetics of donor-derived bacteria and their mechanistic implications in the recipients are still incompletely understood. Humanizing disease models in conventional mice is scalable and offers a complementary approach to germ-free mouse studies. Our results, though admittedly on a limited sample size, build on the notion that that ABX cleanout in conventional mice followed by FMT from a human donor is a viable option. It will also allow the study of engraftment of donor bacteria from both a pharmacokinetic (taxonomy) as well as a pharmacodynamic (metabolites) perspective. Careful consideration must be given to the choice of ABX-regimen and frequency of FMT dosing as they have significant downstream effects in the recipient mice. This model could be used in future studies to study phenotypic effects of FMT from healthy vs. disease donors, as well as to study the efficacy of microbiome-modulators to alter disease phenotypes. Learnings from such studies could also provide valuable guidance as this class of treatment progresses in the clinic.

## Data Availability

All raw microbiome data are available from the SRA (sequence read archive) under accession number PRJNA732891 (https://www.ncbi.nlm.nih.gov/sra/PRJNA732891, accessed on 4 June 2021).

## Supplementary Materials

The following are available online at https://www.mdpi.com/article/10.3390/microorganisms9071399/s1, Table S1. TIC normalized metabolite abundances per sample. Figure S1. Representative phred score plots of Read 1 after quality filtering using the DADA2 algorithm. Figure S2. Representative phred score plots of Read 2 after quality filtering using the DADA2 algorithm. Figure S3. Representative DADA2 model error plots. Figure S4. (A) Box plots of longitudinal changes in richness over time (*n* = 5 mice/group; p-value by Kruskal–Wallis test). PCOA plot of unweighted UniFrac beta-diversity distances in (B) G1, (C) G2, (D) G4 and (E) G5 mice (*n* = 5 mice/group; *p*-value by ANOSIM with 999 permutations). Figure S5. Line plot to compare changes in ‘human’ and ‘mouse’ ASV abundances over time in (C) G2 and (D) G5 mice (*n* = 5 mice/group). The mean and standard deviation of ASV abundances is represented at each time point. *p*-value by one-sided paired Wilcoxon signed-rank test, and adjustment for multiple correction by FDR. Figure S6. Line plot to compare changes in ‘human’ (only those uniquely derived from input material) and ‘mouse’ ASV abundances over time in (A) G2, (B) G3, (C) G5 and (D) G6 mice (*n* = 5 mice/group). The mean and standard deviation of ASV abundances is represented at each time point. p-value by one-sided paired Wilcoxon signed-rank test, and adjustment for multiple correction by FDR. Figure S7. Stacked bar plot showing engraftment of only those phyla that were uniquely derived from the input material in (A) G2 and (B) G5 recipient mice. Figure S8. Stacked bar plot showing engraftment of only those families that were uniquely derived from the input material in (A) G2, (B) G3, (C) G5, and (D) G6 recipient mice. Figure S9. Stacked bar plot of the overall taxonomic landscape at the phylum level of the input material and recipient mice over time in (A) G1, (B) G2, (C) G4, and (D) G5 mice. *n* = 5 mice/group. Only the most abundant phyla are shown. Figure S10. Stacked bar plot of the overall taxonomic landscape at the family level of the input material and recipient mice over time in mice receiving 3-day ABX. (A) G1, (B) G2, and (C) G3 mice. *n* = 5 mice/group. Only the most abundant families are shown. Figure S11. Stacked bar plot of the overall taxonomic landscape at the family level of the input material and recipient mice over time in (A) G4, (B) G5, and (C) G6 mice. *n* = 5 mice/group. Only the most abundant families are shown. Figure S12. Validation experiment in BKS mice using the G6 regimen (3w ABX + 5-FMT). (A) Stacked bar plot showing engraftment of phyla uniquely derived from the input material. Stacked bar plot comparing the most abundant (B) phyla and (C) families in the input material and recipient mice over time.
