# Supplementary material for: Engraftment of Bacteria after Fecal Microbiota Transplantation Is Dependent on Both Frequency of Dosing and Duration of Preparative Antibiotic Regimen
Source: Microorganisms. 2021 Jun 29;9(7):1399. doi: 10.3390/microorganisms9071399 (PMC8306289; doi:10.3390/microorganisms9071399)
Supplement: Supplementary file 1 [file microorganisms-09-01399-s001.zip › Compiled_supplemental_figures_proofs.pdf]

Figure S1

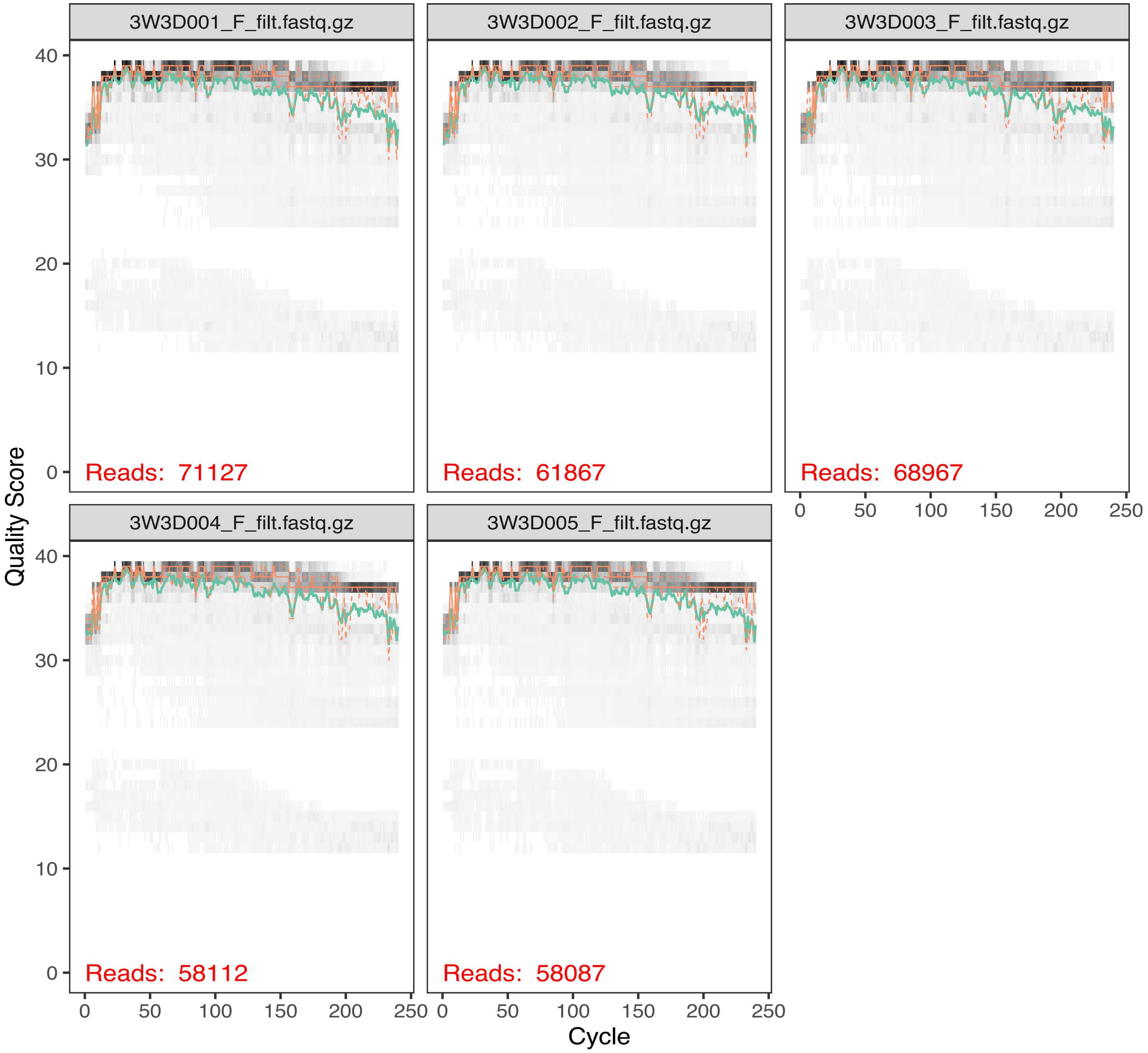
3W3D001_F_filt.fastq.gz
3W3D002_F_filt.fastq.gz
3W3D003_F_filt.fastq.gz
3W3D004_F_filt.fastq.gz
3W3D005_F_filt.fastq.gz
Reads: 71127
Reads: 61867
Reads: 68967
Reads: 58112
Reads: 58087
Quality Score
Cycle
0
10
20
30
40
0
50
100
150
200
250

Figure S2

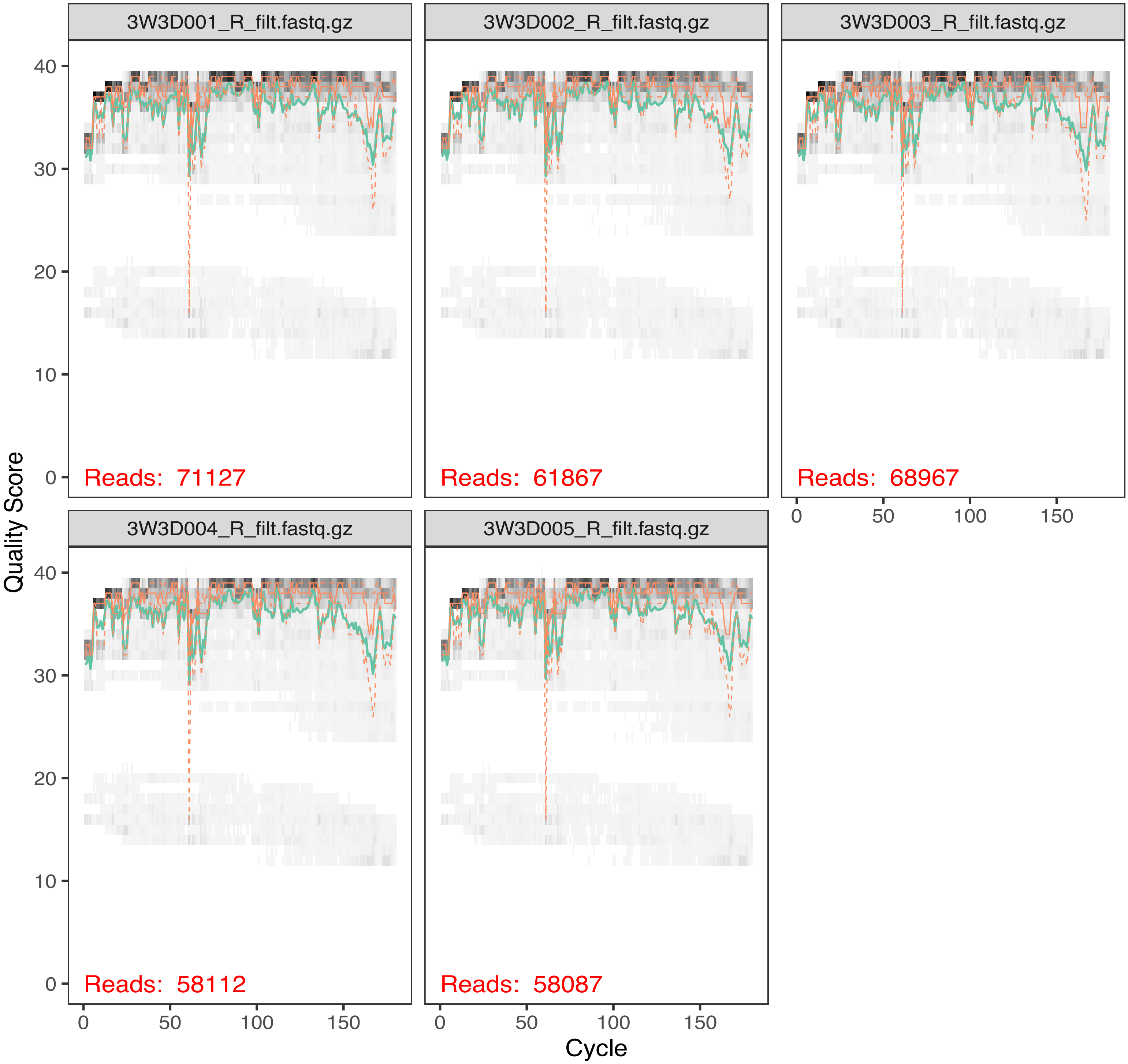
3W3D001_R_filt.fastq.gz
3W3D002_R_filt.fastq.gz
3W3D003_R_filt.fastq.gz
3W3D004_R_filt.fastq.gz
3W3D005_R_filt.fastq.gz
Reads: 71127
Reads: 61867
Reads: 68967
Reads: 58112
Reads: 58087
Quality Score
Cycle
0
10
20
30
40
0
50
100
150

Figure S3

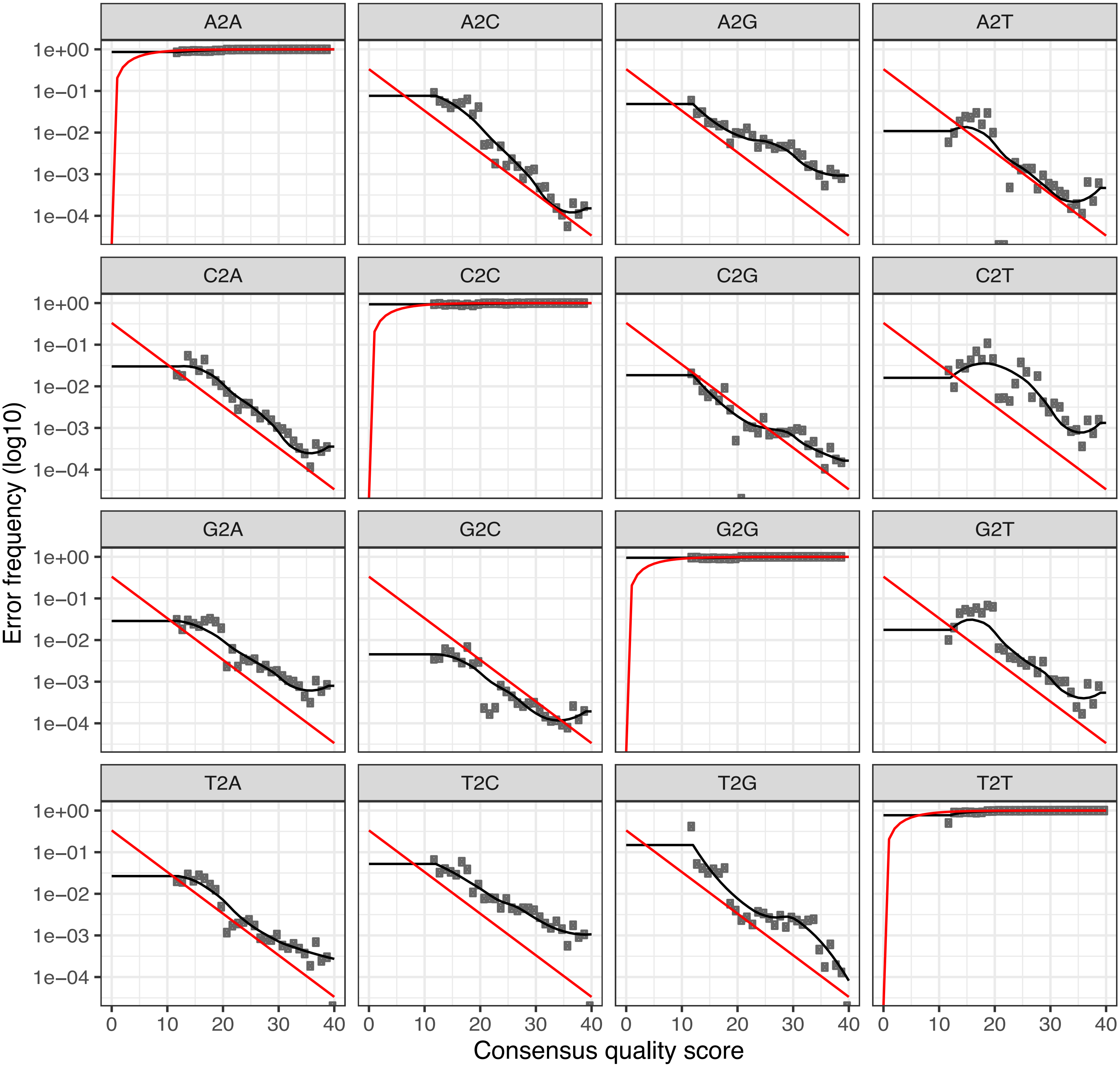

A2A
A2C
A2G
A2T
C2A
C2C
C2G
C2T
G2A
G2C
G2G
G2T
T2A
T2C
T2G
T2T
1e+00
1e−01
1e−02
1e−03
1e−04
Error frequency (log10)
0
10
20
30
40
Consensus quality score

Figure S4

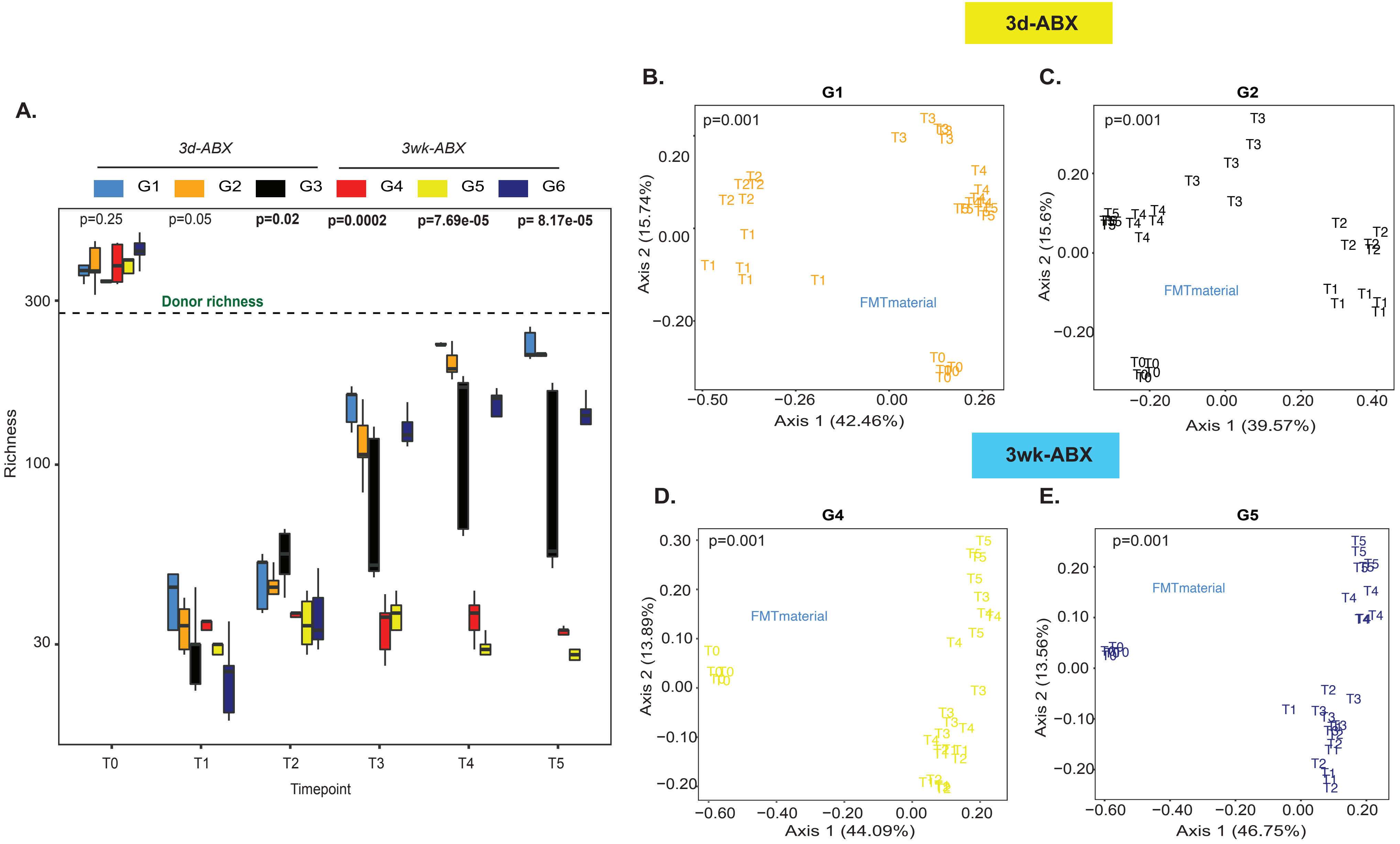

A.
3d-ABX
3wk-ABX
G1
G2
G3
G4
G5
G6
p=0.25
p=0.05
p=0.02
p=0.0002
p=7.69e-05
p= 8.17e-05
Donor richness
Richness
300
100
30
T0
T1
T2
T3
T4
T5
Timepoint
3d-ABX
B.
G1
p=0.001
FMTmaterial
Axis 2 (15.74%)
Axis 1 (42.46%)
C.
G2
p=0.001
FMTmaterial
Axis 2 (15.6%)
Axis 1 (39.57%)
3wk-ABX
D.
G4
p=0.001
FMTmaterial
Axis 2 (13.89%)
Axis 1 (44.09%)
E.
G5
p=0.001
FMTmaterial
Axis 2 (13.56%)
Axis 1 (46.75%)

Figure S5

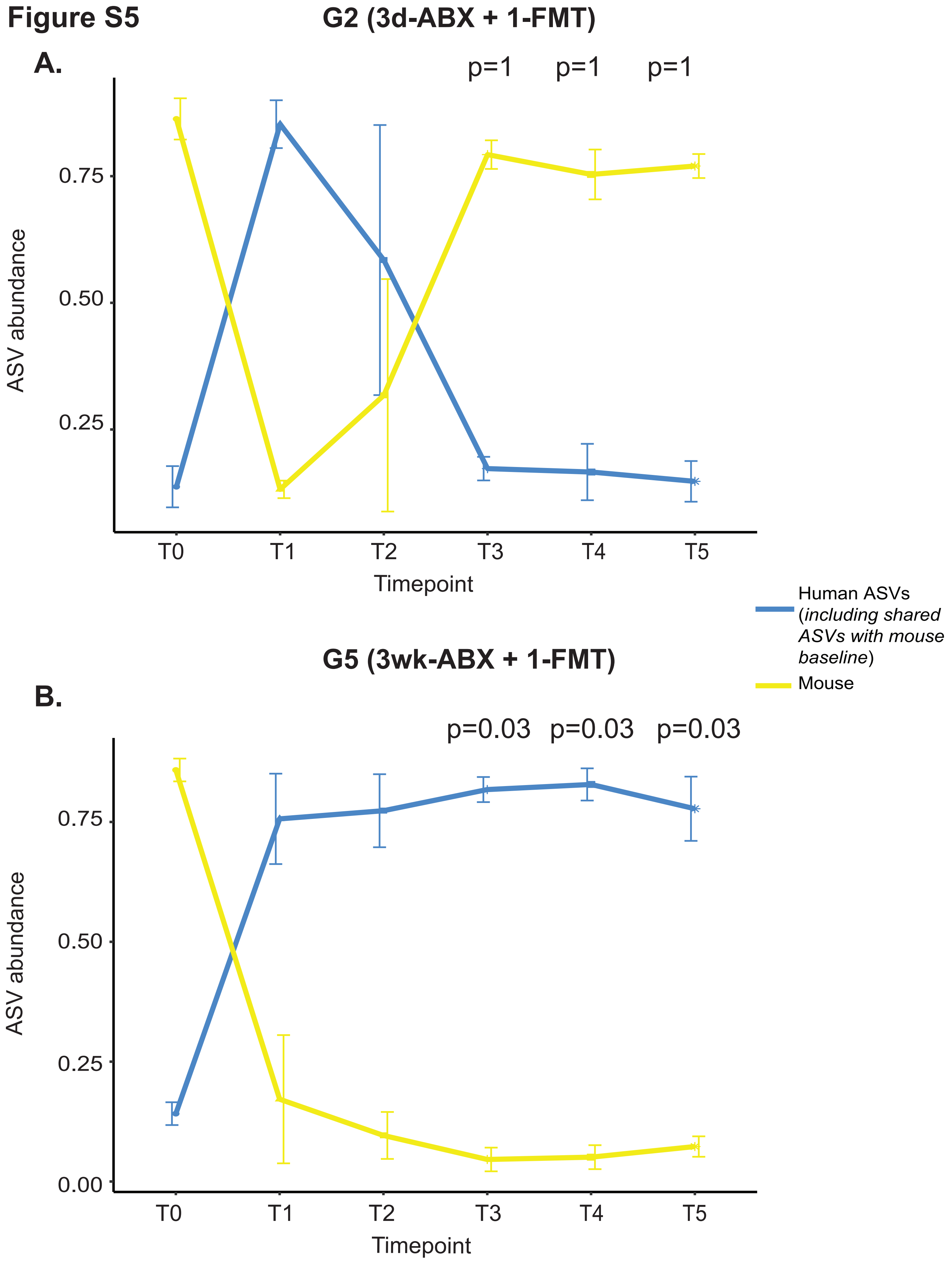

G2 (3d-ABX + 1-FMT)
A.
p=1
p=1
p=1
0.75
0.50
0.25
ASV abundance
T0
T1
T2
T3
T4
T5
Timepoint
Human ASVs (including shared ASVs with mouse baseline)
Mouse
G5 (3wk-ABX + 1-FMT)
B.
p=0.03
p=0.03
p=0.03
0.75
0.50
0.25
0.00
ASV abundance
T0
T1
T2
T3
T4
T5
Timepoint

Figure S6

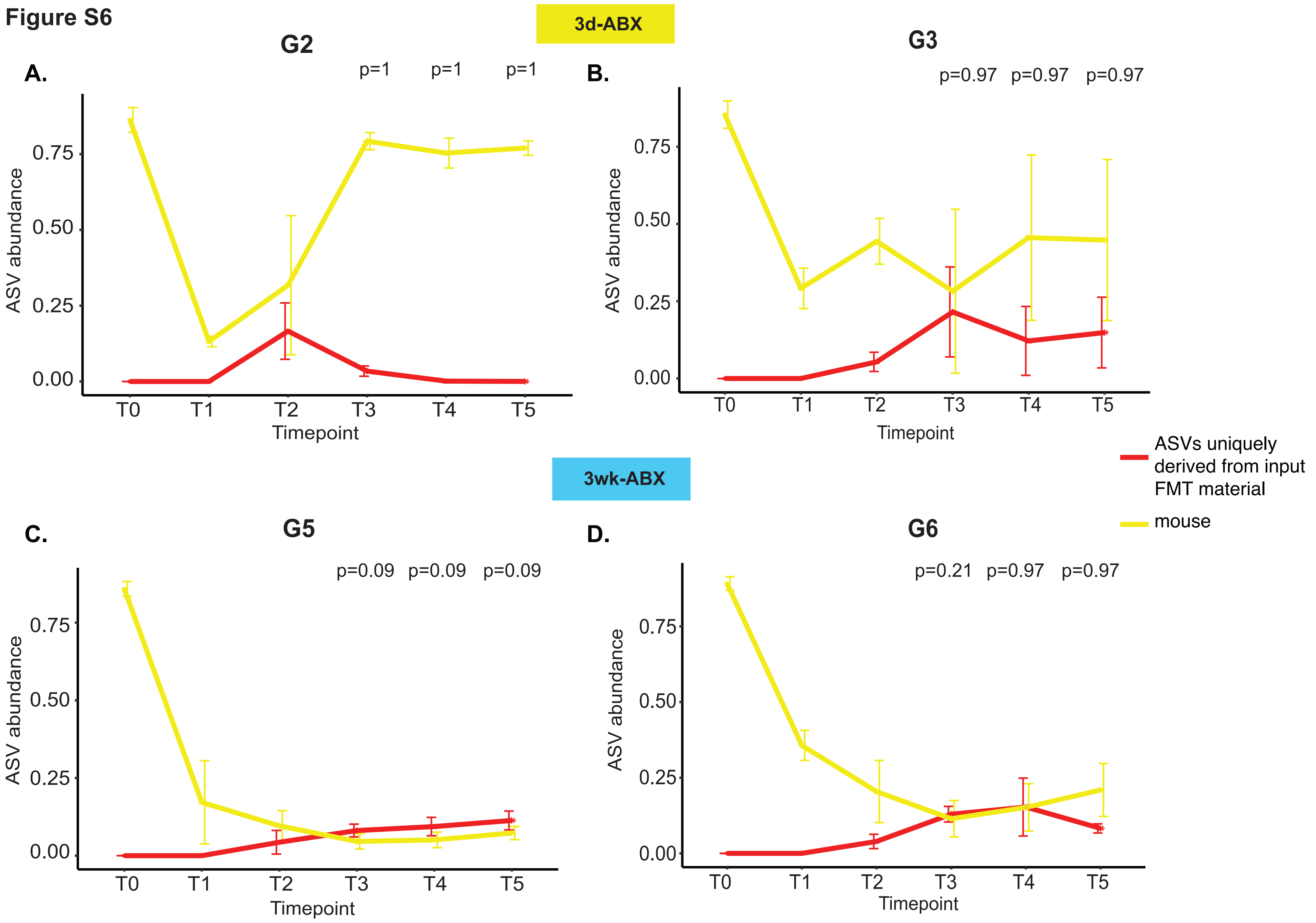

3d-ABX
G2
A.
p=1 p=1 p=1
ASV abundance
0.75
0.50
0.25
0.00
T0 T1 T2 T3 T4 T5
Timepoint
G3
B.
p=0.97 p=0.97 p=0.97
ASV abundance
0.75
0.50
0.25
0.00
T0 T1 T2 T3 T4 T5
Timepoint
ASVs uniquely derived from input FMT material
mouse
3wk-ABX
C.
G5
p=0.09 p=0.09 p=0.09
ASV abundance
0.75
0.50
0.25
0.00
T0 T1 T2 T3 T4 T5
Timepoint
D.
G6
p=0.21 p=0.97 p=0.97
ASV abundance
0.75
0.50
0.25
0.00
T0 T1 T2 T3 T4 T5
Timepoint

Figure S7

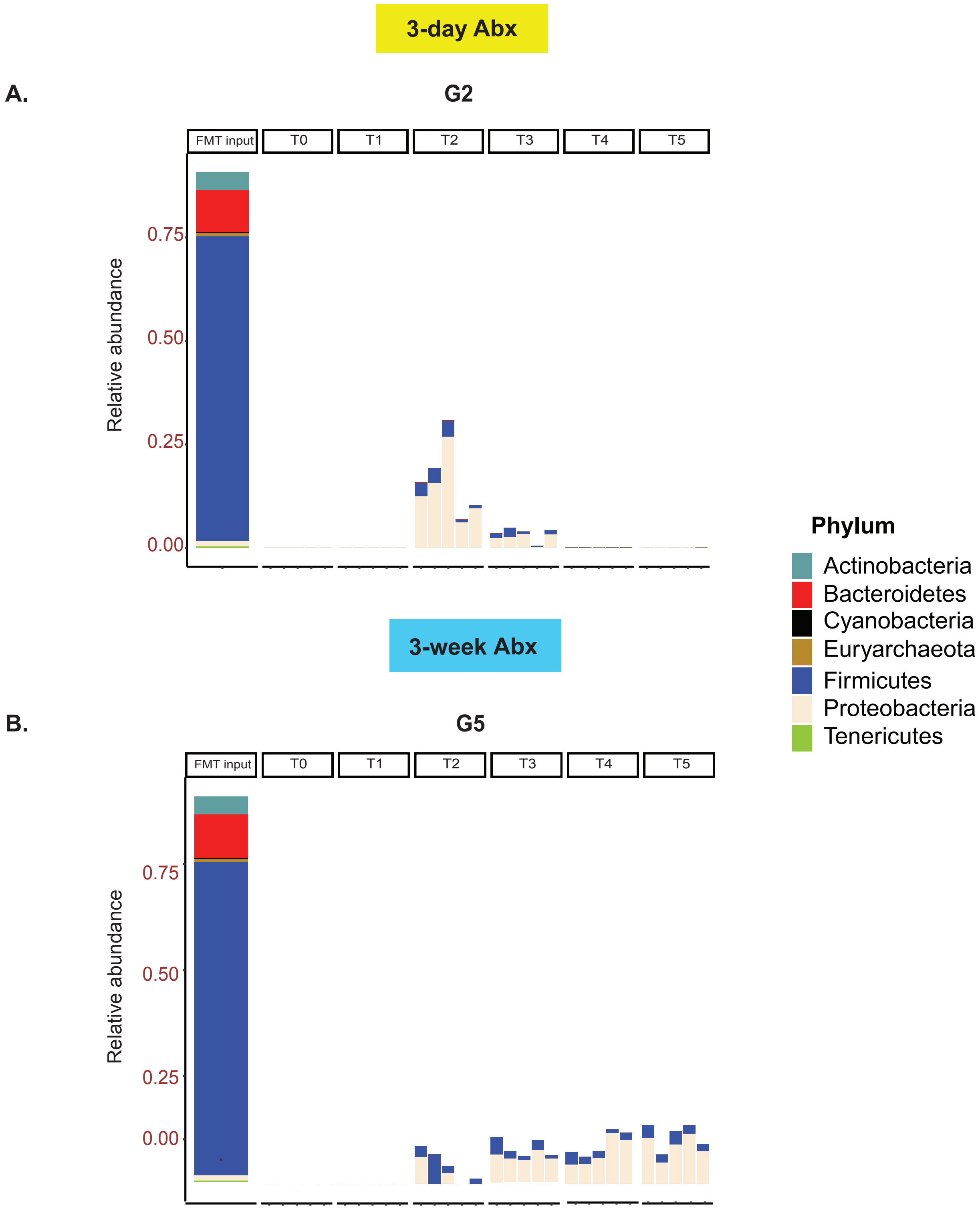
3-day Abx
A.
G2
FMT input
T0
T1
T2
T3
T4
T5
Relative abundance
0.75
0.50
0.25
0.00
3-week Abx
B.
G5
FMT input
T0
T1
T2
T3
T4
T5
Relative abundance
0.75
0.50
0.25
0.00
Phylum
Actinobacteria
Bacteroidetes
Cyanobacteria
Euryarchaeota
Firmicutes
Proteobacteria
Tenericutes

Figure S8

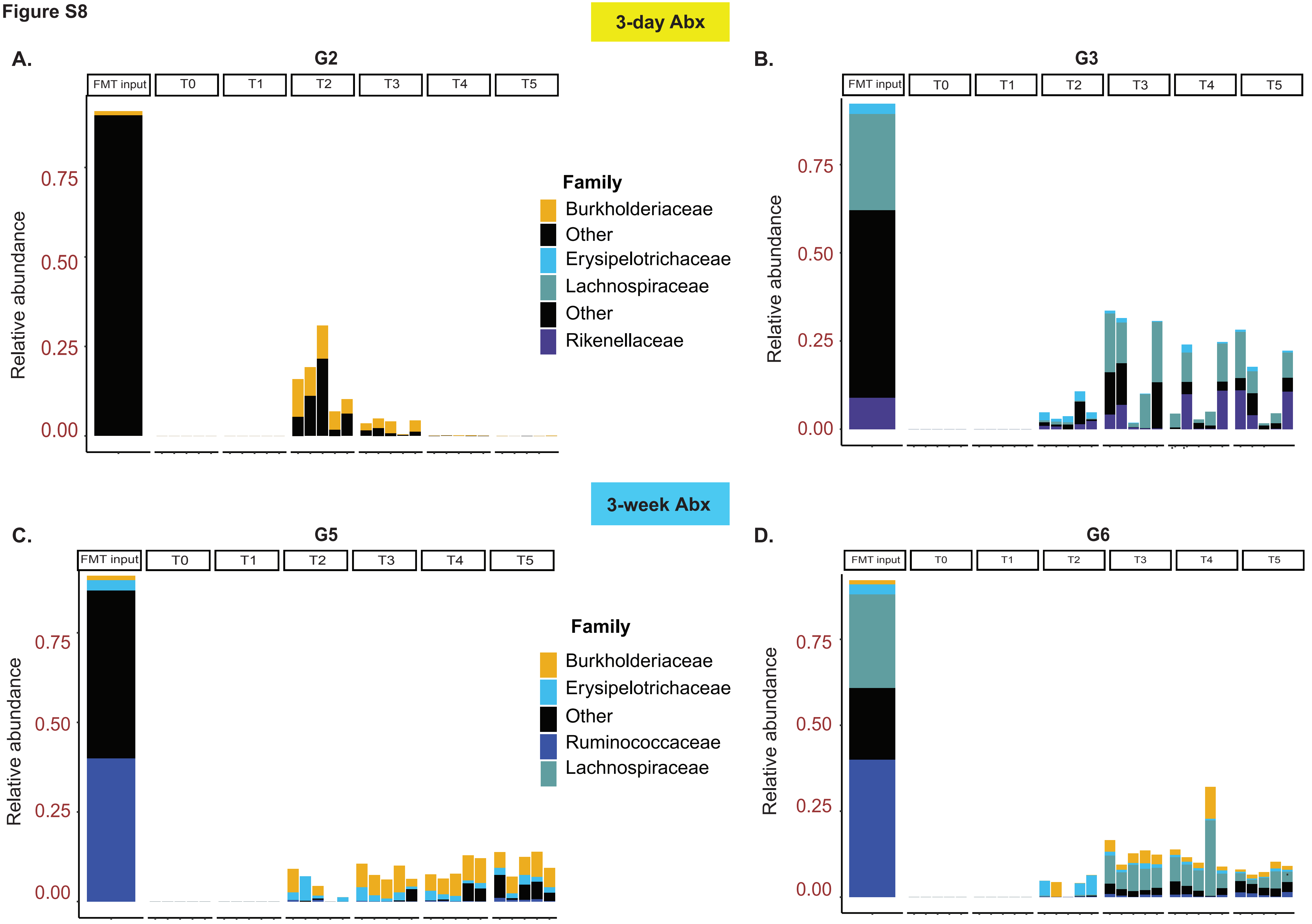

3-day Abx
A.
G2
FMT input
T0
T1
T2
T3
T4
T5
Relative abundance
0.00
0.25
0.50
0.75
Family
Burkholderiaceae
Other
Erysipelotrichaceae
Lachnospiraceae
Other
Rikenellaceae
B.
G3
FMT input
T0
T1
T2
T3
T4
T5
Relative abundance
0.00
0.25
0.50
0.75
3-week Abx
C.
G5
FMT input
T0
T1
T2
T3
T4
T5
Relative abundance
0.00
0.25
0.50
0.75
Family
Burkholderiaceae
Erysipelotrichaceae
Other
Ruminococcaceae
Lachnospiraceae
D.
G6
FMT input
T0
T1
T2
T3
T4
T5
Relative abundance
0.00
0.25
0.50
0.75

Figure S9

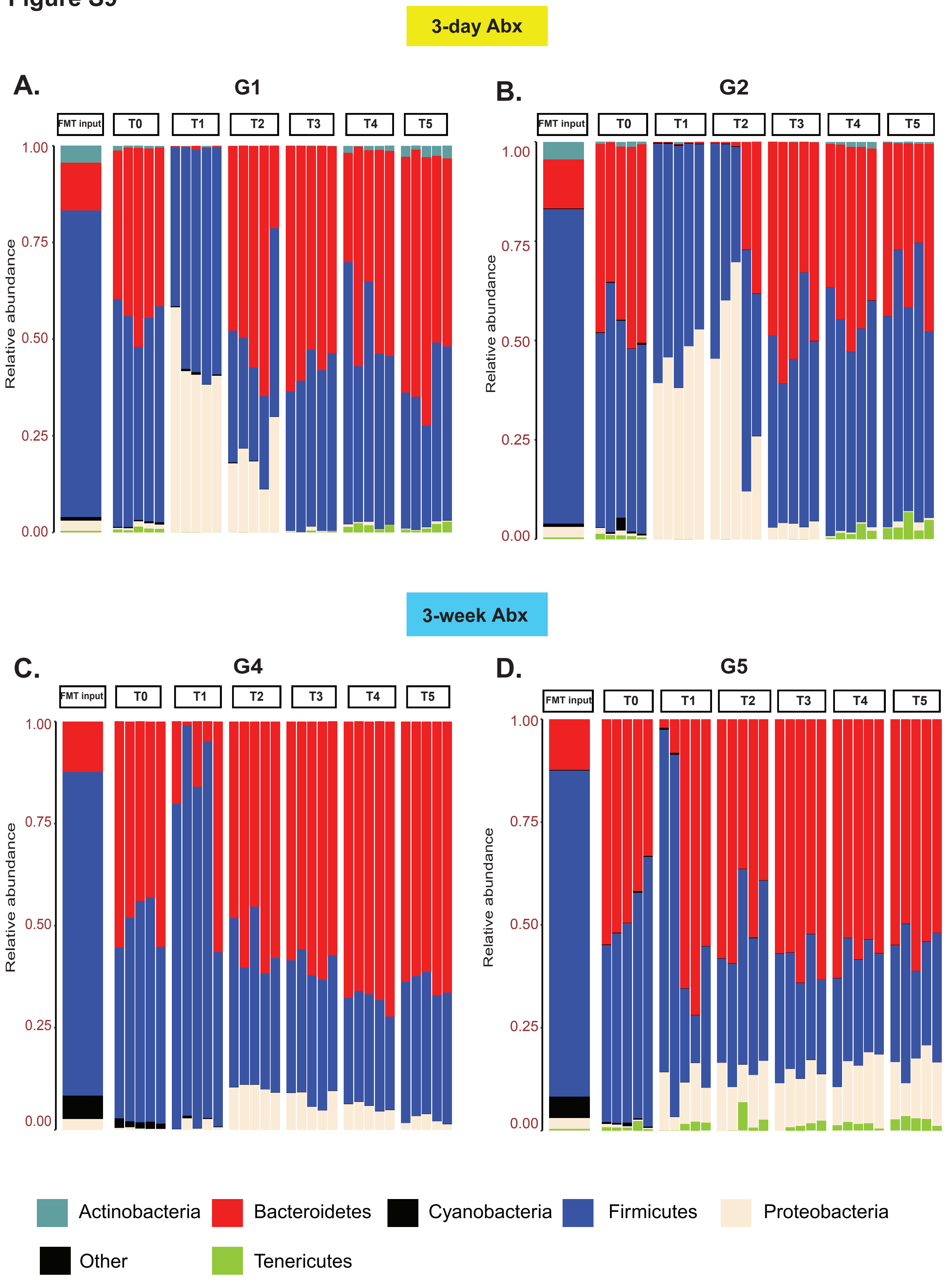
3-day Abx
A.
G1
B.
G2
FMT input
T0
T1
T2
T3
T4
T5
Relative abundance
1.00
0.75
0.50
0.25
0.00
3-week Abx
C.
G4
D.
G5
Actinobacteria
Bacteroidetes
Cyanobacteria
Firmicutes
Proteobacteria
Other
Tenericutes

Figure S10

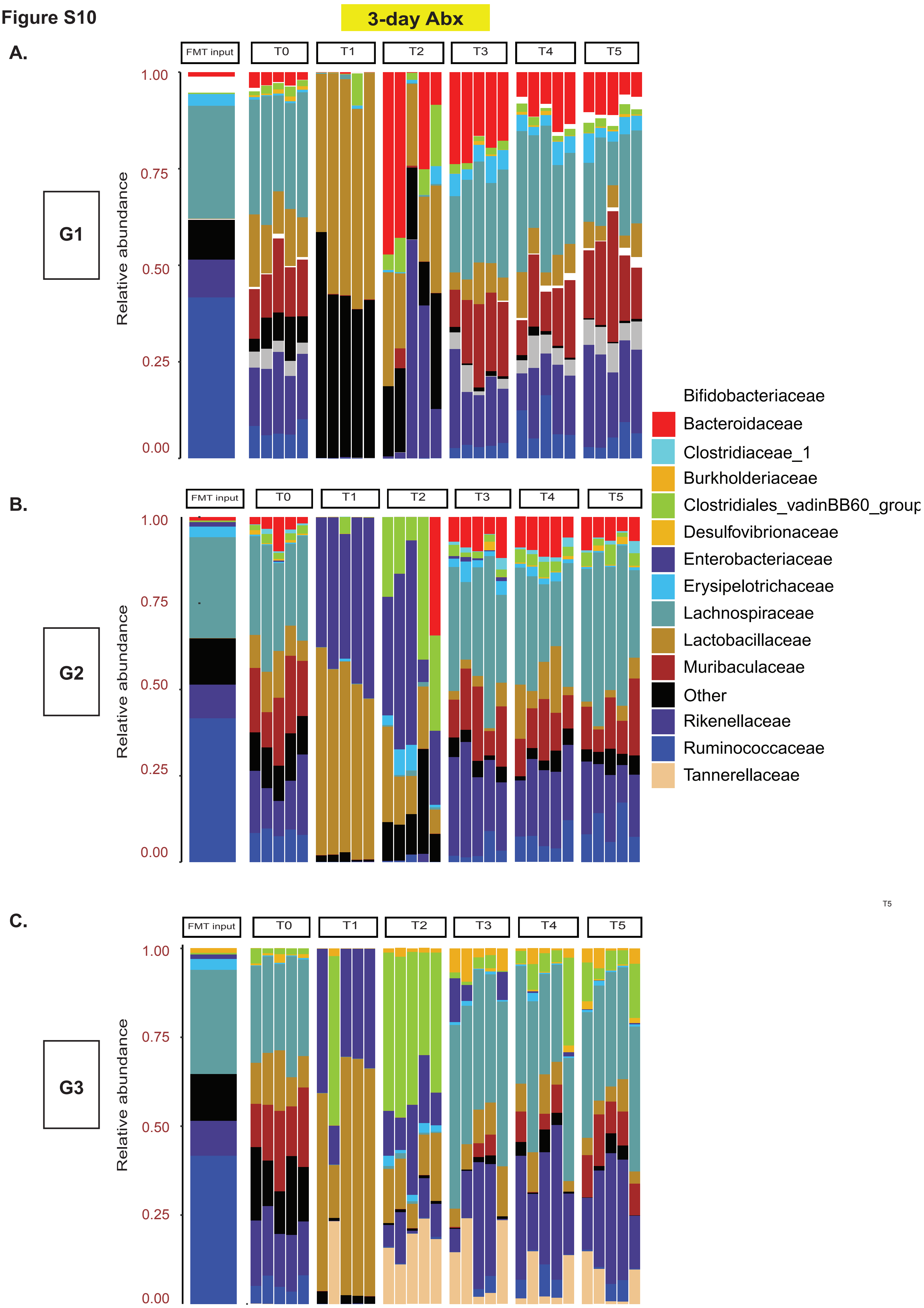

3-day Abx
A.
B.
C.
G1
G2
G3
FMT input
T0
T1
T2
T3
T4
T5
Relative abundance
1.00
0.75
0.50
0.25
0.00
Bifidobacteriaceae
Bacteroidaceae
Clostridiaceae_1
Burkholderiaceae
Clostridiales_vadinBB60_group
Desulfovibrionaceae
Enterobacteriaceae
Erysipelotrichaceae
Lachnospiraceae
Lactobacillaceae
Muribaculaceae
Other
Rikenellaceae
Ruminococcaceae
Tannerellaceae

Figure S11

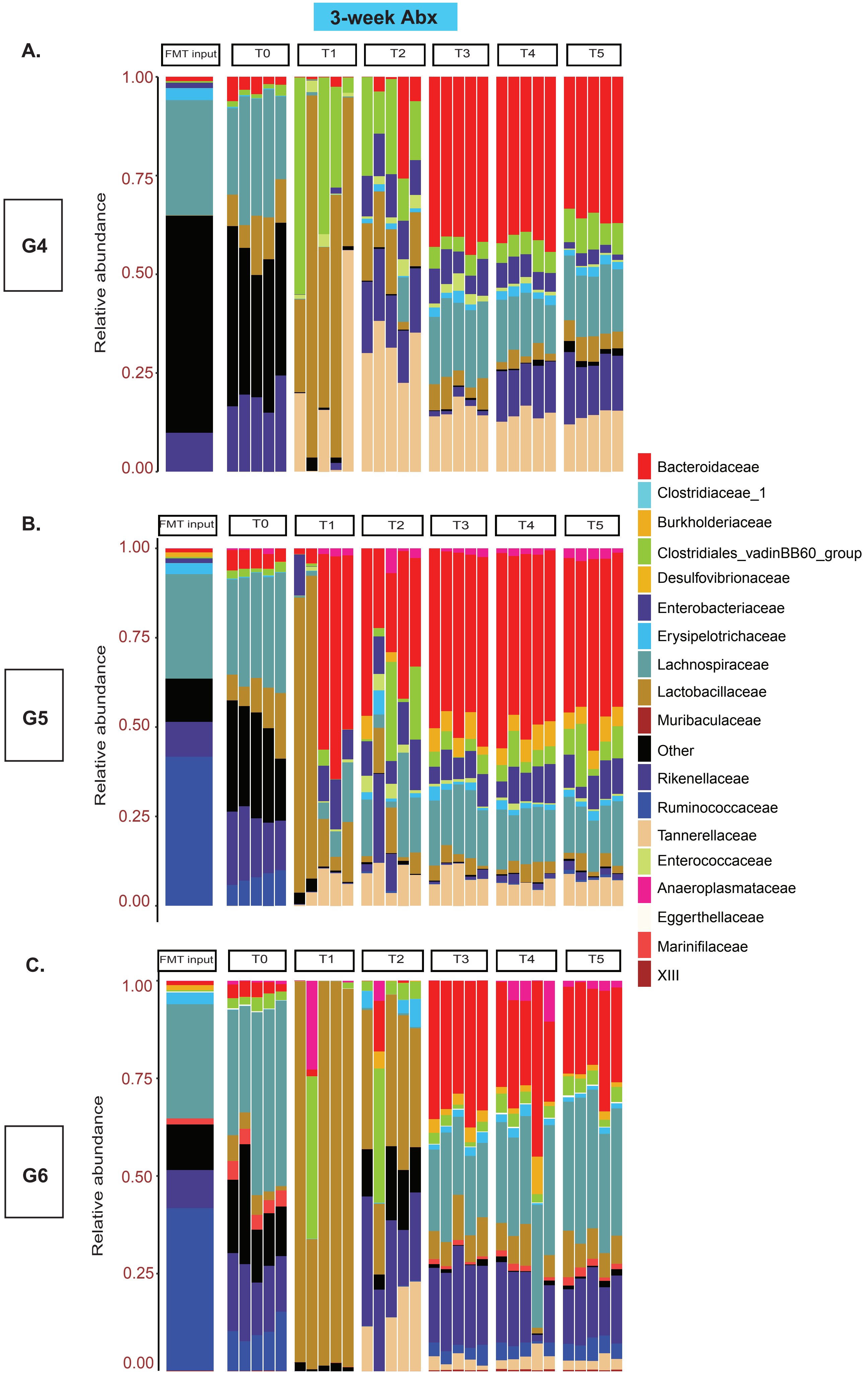

3-week Abx
A.
G4
B.
G5
C.
G6
FMT input
T0
T1
T2
T3
T4
T5
Relative abundance
1.00
0.75
0.50
0.25
0.00
Bacteroidaceae
Clostridiaceae_1
Burkholderiaceae
Clostridiales_vadinBB60_group
Desulfovibrionaceae
Enterobacteriaceae
Erysipelotrichaceae
Lachnospiraceae
Lactobacillaceae
Muribaculaceae
Other
Rikenellaceae
Ruminococcaceae
Tannerellaceae
Enterococcaceae
Anaeroplasmataceae
Eggerthellaceae
Marinifilaceae
XIII

Figure S12

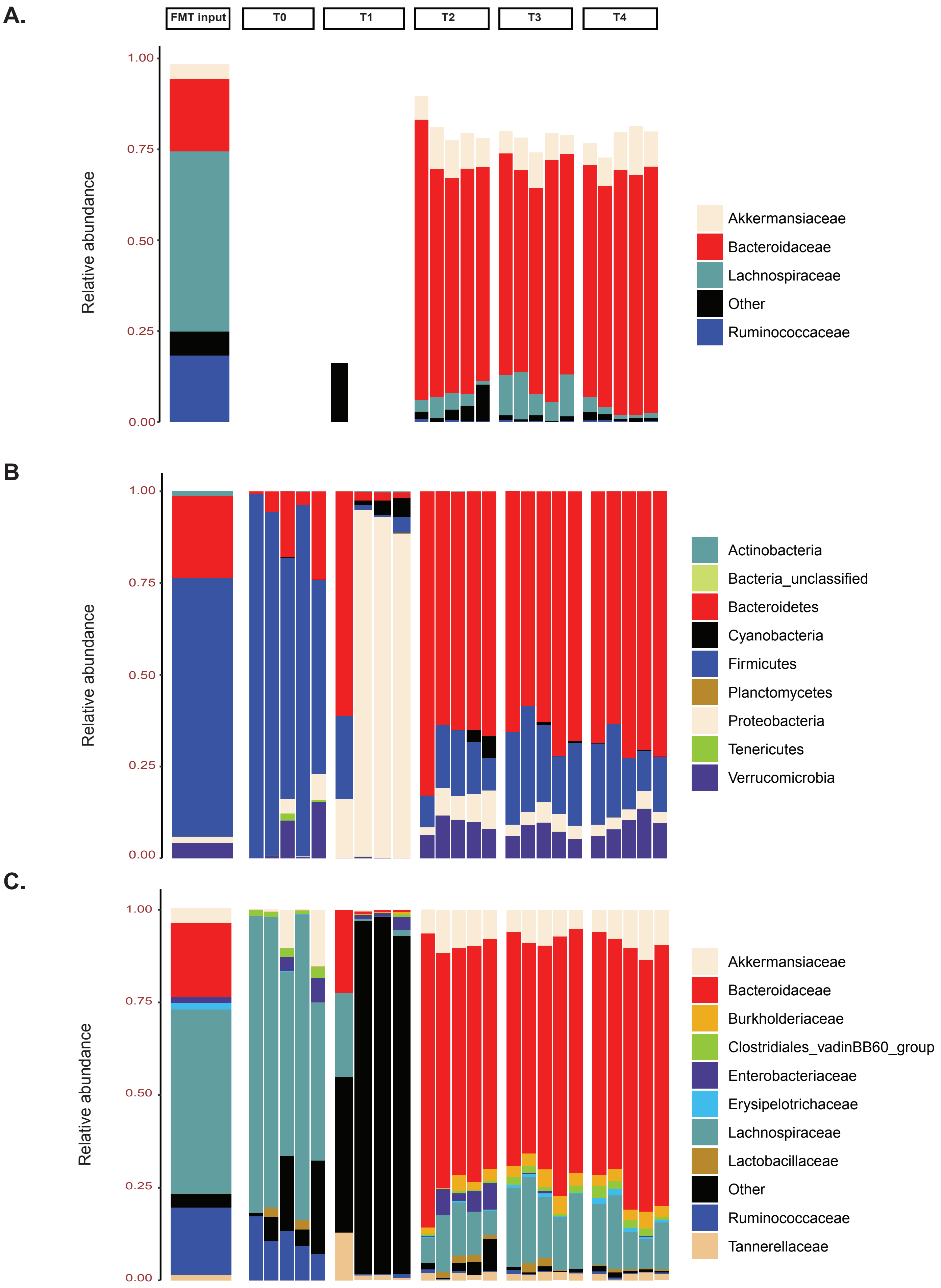
A.
FMT input
T0
T1
T2
T3
T4
Relative abundance
1.00
0.75
0.50
0.25
0.00
Akkermansiaceae
Bacteroidaceae
Lachnospiraceae
Other
Ruminococcaceae
B
Relative abundance
1.00
0.75
0.50
0.25
0.00
Actinobacteria
Bacteria_unclassified
Bacteroidetes
Cyanobacteria
Firmicutes
Planctomycetes
Proteobacteria
Tenericutes
Verrucomicrobia
C.
Relative abundance
1.00
0.75
0.50
0.25
0.00
Akkermansiaceae
Bacteroidaceae
Burkholderiaceae
Clostridiales_vadinBB60_group
Enterobacteriaceae
Erysipelotrichaceae
Lachnospiraceae
Lactobacillaceae
Other
Ruminococcaceae
Tannerellaceae
